# Processing of Grape Bagasse and Potato Wastes for the Co-Production of Bacterial Cellulose and Gluconic Acid in an Airlift Bioreactor

**DOI:** 10.3390/polym15193944

**Published:** 2023-09-29

**Authors:** Manuel Vázquez, Gema Puertas, Patricia Cazón

**Affiliations:** Department of Analytical Chemistry, Faculty of Veterinary, Campus Terra, University of Santiago de Compostela, 27002 Lugo, Spain

**Keywords:** bacterial cellulose, gluconic acid, hydrolysis, food waste, airlift fermentation

## Abstract

The feasibility of using Garnacha Tintorera bagasse and potato wastes as substrate for the co-production of bacterial cellulose (BC) and gluconic acid by *Komagataibacter xylinus* fermentation was studied. Firstly, the sulfuric acid hydrolysis of bagasse was evaluated depending on the sulfuric acid concentration (2–4%), temperature (105–125 °C), and time (60–180 min). The bagasse hydrolysates showed a low monosaccharide concentration profile: glucose 3.24–5.40 g/L; cellobiose 0.00–0.48 g/L; arabinose 0.66–1.64 g/L and xylose 3.24–5.40 g/L. However, the hydrolysis treatment enhanced the total phenolic content of the bagasse extract (from 4.39 up to 12.72 mg GAE/g dried bagasse). The monosaccharide profile of the culture medium was improved by the addition of potato residues. From a medium containing bagasse–potato powder (50:50 *w*/*w*) and optimal hydrolysate conditions (125 °C for 60 min and 2% H_2_SO_4_), the composition of glucose increased up to 30.14 g/L. After 8 days of fermentation in an airlift bioreactor by *Komagataibacter xylinus*, 4 g dried BC/L and 26.41 g gluconic acid/L were obtained with a BC productivity of 0.021 g/L·h, an efficiency of 0.37 g/g and yield of 0.47 g/g. The productivity of gluconic acid was 0.14 g/L·h with an efficiency of 0.93 g/g and yield of 0.72 g/g. This research demonstrates the promising potential of utilizing waste materials, specifically Garnacha Tintorera bagasse and potato residues, as sustainable substrates for the co-production of valuable bioproducts, such as bacterial cellulose and gluconic acid.

## 1. Introduction

EU-wide monitoring revealed that in 2020, nearly 59 million tons of food waste was produced in the EU, equivalent to an average of 131 kg per inhabitant with a market value of 132 billion euros (source: https://food.ec.europa.eu/safety/food-waste_en (accessed on 4 September 2023)). This figure only accounts for food waste at the retail and consumption levels, excluding losses from on-farm harvesting, transport, storage, wholesale, and processing. Therefore, the real value of agri-food waste generated each year is much higher. A prominent solution to reduce food loss and waste is the extraction and/or bioconversion of food wastes into value-added materials for their application in several emerging fields [1]. For instance, food wastes can be used directly without separation or purification processes as a source of nutrients for biotechnological routes and fermentations to produce bio-based polymers or chemical products [2]. Among the agri-food wastes that are produced in large quantities, and they could be an interesting source of bioactive compounds or carbon sources, are grape bagasse and discarded potatoes.

Grape bagasse from *Vitis vinifera* is the main by-product of wine production and is available worldwide in large quantities (up to 1.34 × 10^10^ kg of fresh weight per year) [3,4]. Grape bagasse is the remaining solid after fermentation and pressing, which contains the skins, pulp, seeds, and stems of the grape [5]. Despite removing most fermentable sugars after the pressing process, grape bagasse contains important active compounds, such as phenols, which could be of interest for enriching growing media and improving yields in some biotechnological processes. On the other hand, potato (*Solanum tuberosum*) is the fourth leading crop after rice, wheat, and maize, with a global total production of 3.76 × 10^11^ kg in 2021 (www.FAO.org (accessed on 4 September 2023)). As previous work has shown, a high content of fermentable sugars can be obtained by non-commercial potato hydrolysis [6]. Due to the large amount of waste generated from grape bagasse and discarded potato and its complementary composition that can result in a low-cost enriched culture medium, it may represent an alternative for the co-production of bacterial cellulose (BC) and gluconic acid.

Bacterial cellulose (BC) has garnered significant research attention over the past decades, driven by its biodegradability and versatile potential applications across various fields. [7,8]. The main advantage of BC over vegetable cellulose is that BC is synthesized in a pure way, free of lignin, hemicellulose, or pectin. Hence, the purification process of BC does not imply expensive processes and the use of environmentally hazardous chemicals [7]. The relevance of BC is reflected in its global market. It is estimated to reach USD 771.76 Million by 2027, growing at a compound annual growth rate of 12.6% between 2019 and 2027 (www.profsharemarketresearch.com (accessed on 4 September 2023)). However, the large-scale industrialization and commercialization of BC remains a challenge due to high fermentation costs, low productivity, and expensive culture media. Low-cost culture media from large waste of food-derived biomass have been evaluated for BC production to overcome this handicap [9,10]. Wine wastes such as grape pomace extract combined with corn steep liquor were studied as culture media for BC [11]. Additionally, other works also evaluated wine industry wastes as carbon sources for BC productions, like grape skin aqueous extract combined with cheese whey [12], white and red grape bagasse [13], and grape juice with cane sugar [14]. Other waste from the fruit industry evaluated were citrus peel [15,16], orange peel [17], wastewater of candied jujube [18], pineapple, apple, and Japanese pear [19], among others. However, the acid hydrolysis of whole grape bagasse without a previous splitting of the parts (skin, seed, stalk, pulp) mixed with potato has not been evaluated.

On the other hand, grape bagasse and potato can be bio-converted into D-gluconic acid. D-gluconic acid or pentahydroxycaproic acid (C_6_H_12_O_7_) is usually produced by microbial oxidation of glucose. The alkali salts of gluconic acid (calcium gluconate and sodium gluconate) are extensively used in chemical, pharmaceutical, food, beverages, and construction industries, being a multifunctional carbonic acid [20]. Due to the multiple applications of acid gluconic in different industries, its demand is steadily increasing globally. The global gluconic acid market is expected to reach USD 1.9 billion by 2028, growing at a compound annual growth rate of 5% during the forecast period 2021–2028 (https://www.fiormarkets.com/ (accessed on 5 September 2023)). The average cost of production of gluconic acid is USD 1.20/kg for calcium gluconate [21]. Consequently, its huge consumption in the market has raised interest in the development of an efficient and economical system for the production of gluconic acid [21,22]. Carbon sources like potato waste [22], grape must [21,23], sugarcane molasses [21], and banana must [24] have been evaluated as nutrients for the production of gluconic acid.

Additionally, the co-production of two or more value-added products is an interesting approach to improving the economic viability of bioprocesses. In fact, this is more interesting when the co-production is by using food waste as a substrate source to reduce the production cost [25,26]. Several examples of co-production of gluconic acid and different compounds can be found in the literature, such as the simultaneous production of BC and pear vinegar by fermentation of pear peel and pomace [27], 5-hydroxymethyl furfural and gluconic acid [25], single cell oil and gluconic acid [26], gluconic acid and green hydrogen [28] or gluconic acid and xylonic acid [29].

Therefore, the objective of this work is double. First, the optimization of the operational conditions for the acid hydrolysis of whole Garnacha Tintorera grape bagasse without a previous splitting of the parts (skin, seed, stalk, pulp) to obtain an extract with a high concentration of phenolic compounds and the minimum concentration of microbial growth inhibitors. Then, to study the co-production of BC and gluconic acid by *Komagateibacter xylinus* in an airlift reactor using the acid hydrolysates of grape bagasse as a source of antioxidants and potato waste as a source of fermentable sugars.

## 2. Materials and Methods

### 2.1. Chemicals and Standards

*Komagateibacter xylinus* as a pure freeze-dried culture was obtained from “Colección Española de Cultivos Tipo” (CECT) (Paterna, Spain). Grape bagasse from *Vitis vinifera* L. Garnacha Tintorera, which contains skins, pulp, seeds, and stems, was obtained after the pressing process in winemaking. The bagasse raw material was kindly provided by a local winery (42°33′58.2″ N 7°40′37.4″ W) in the Ribeira Sacra region (Lugo, Spain). Potato samples (*Solanum tuberosum*) were gently supplied by a local company called Pitita’s Farm (Dozón, Spain). They were harvested at the geocoordinates 42°36′14.0″ N 8°01′24.6″ W.

Yeast extract, methanol, ethanol, sulfuric acid (95–97%), and sodium carbonate were supplied by Scharlau Microbiology (Barcelona, Spain). Standards of gluconic acid, D(+)-glucose monohydrate (99%), D-xylose (99%), L(+)-arabinose (99%), 5-hydroxymethyl furfural (HMF) (98%) and furfural (99%) standards were purchased from Acros organics (Geel, Belgium). Analytical HPLC grade reagents and solvents were exclusively employed for the HPLC analysis. Folin–Ciocalteu reagents were supplied by Panreac (Barcelona, Spain).

### 2.2. Grape Bagasse and Discarded Potato Pre-Treatments

The grape bagasse (skins, pulp, seeds, and stems) and potato were dried in a hot air dryer at 50 °C for 24 h. The dried grape bagasse was pulverized into powder and filtered using a 0.5 mm mesh sieve. The resulting uniform sample, with particles smaller than 0.5 mm, was then stored in an airtight container at room temperature for future use. The same procedure was applied to the potatoes after drying, washing, and slicing.

Aliquots of the homogenized raw materials were analyzed for moisture determination, drying a known amount of sample at 105 °C to constant weight. The moisture content test was carried out by triplicate. Additionally, the water activity of the Garnacha bagasse powder was determined by triplicate with a water activity measuring equipment, Aqualab^®^ (METER Group, Pullman, WA, USA).

### 2.3. Acid Hydrolysis Treatment of Grape Bagasse

An autoclave was utilized to carry out the sulfuric acid hydrolysis. A specific amount of bagasse powder was placed in 0.5 L bottles by adding a solution of sulfuric acid until a final concentration ranged between 2–4% and a solid/liquid ratio of 1:10. The mixture was kept under vigorous stirring for 1 h to allow all the matter to be wetted and obtain complete supernatant recovery. Then, the reaction mixture was treated at the set hydrolysis conditions of temperature (105–125 °C) and time (60–180 min). The set of experiments followed a Box–Benkhen experimental design using three factors (sulfuric acid concentration, temperature, and time as independent variables) and three levels (−1, 0, 1). The operational conditions were set as shown in Table 1.

Once the heat treatment was completed, the hydrolysate liquors were cooled at room temperature and neutralized with CaCO_3_ until pH 6. The neutralized solutions were filtered through filter paper (10 µm porosity) to remove un-dissolved material, and the CaSO_4_ precipitated.

### 2.4. Analytical Methods

Glucose, gluconic acid, cellobiose, xylose, arabinose, and HMF were determined by HPLC using a Rezex RHM (Phenomenex, Torrance, CA, USA) column with isocratic elution (flow rate of 0.400 mL/min and mobile phase of 0.025 M H_2_SO_4_), a column oven set at 45 °C and a refractive index detector (RI) (LC 2000 plus, Jasco, Tokyo, Japan). RI detector was used for the analysis of glucose, cellobiose, xylose, and arabinose. Gluconic acid was detected at 220 nm, and furfural and HMF were detected at 275 nm by a diode array detector (DAD) [30,31].

The antioxidant capacity of the hydrolyzed grape bagasse and the synthesized BC were determined by the total phenolic content (TPC) expressed as mg of gallic acid equivalents (GAE) per 1 g of dried bagasse or dried BC following the Folin–Ciocalteu method as described elsewhere [32,33].

### 2.5. Culture Media and Bacterial Cellulose Production in Airlift Bioreactor

A mixture of bagasse and potato powder in a 50:50 (*w*/*w*) ratio was prepared in 0.5 L bottles by adding a 2% sulfuric acid solution to achieve a final solid-to-liquid ratio of 1:10. After keeping under vigorous stirring for 1 h, the mixture was heat-treated at 125 °C for 1 h. Subsequently, the hydrolysate liquors were neutralized and filtered using the previously mentioned procedure. The culture media consisting of grape bagasse–potato was prepared using the obtained hydrolysate and supplemented with 10 g of yeast extract per liter.

Pre-inoculum was prepared by triplicate from a starter stock of *K. xylinus* with 100 mL of bagasse–potato culture medium in 250 mL Erlenmeyer flasks previously sterilized at 121 °C for 15 min. The pre-inoculums were incubated under static conditions for 2 days at 30 °C. Afterward, the whole pre-inoculums (300 mL) were placed in a 5 L airlift bioreactor (Minifors, Infors AG, Bottmingen/Basel, Switzerland) filled with fresh, sterilized bagasse–potato culture medium to a final working volume of 3 L. The dry weight (g) of the BC produced at the end of fermentation was determined by weighing samples dried at 105 °C for 24 h, enough time to reach stable weight [34]. BC concentration (in g/L) was calculated by considering the amount of BC synthesized (dry weight, in grams) per unit volume of the airlift bioreactor (3 L). The concentration of gluconic acid (in g/L) was determined by analytical methods described in the previous section. BC and gluconic acid productivity (g/L·h), yield, and efficiency were calculated according to the Equations (1)–(3) [35]:

(1) $$Productivity\;\left(g/L\cdot h\right)=X/V\cdot t$$

(2) $$Yield=\left(X-X_{0}\right)/\left(S_{0}-S\right)$$

(3) $$Efficiency=\left(X-X_{0}\right)/S_{0}$$

where *X* represents the biomass concentration under steady-state conditions (in g/L), *V* is the reaction volume (in liters), *t* is the reaction time (in days), *X*_0_ is the initial biomass concentration (in g/L), *S* is the glucose concentration (in g/L), and *S*_0_ is the initial glucose concentration (in g/L).

### 2.6. Statistical Analysis

Response surface methodology using Box–Behnken design was used to optimize the response of the variables. This design has been employed to examine the relationship between response variables and a set of quantitative experimental parameters based on response surface methodology. Table 2 shows the experiment design. Outliers were identified using Cook’s distance method [36], and the Box-Cox data transformation was used to reduce anomalies [37]. The multifactor analysis of variance (ANOVA) was applied. The statistical analysis was accomplished using Design Expert^®^10.0.6 software (Stat-Ease, Inc., Minneapolis, MN, USA).

## 3. Results

The acid hydrolysis of Garnacha Tintorera grape bagasse was carried out following the conditions indicated in the experimental design (Table 2). The concentration of sulfuric acid ranged from 2 to 4% (*w*/*w*), temperature ranged from 105 to 125 °C, and time ranged from 60 to 180 min. The concentration of glucose, cellobiose, arabinose, xylose, acetic acid, HMF, furfural, and total phenolic compounds (dependent variables) obtained are listed in Table 3. The effect of the sulfuric acid concentration, temperature, and time on the dependent variables was modeled using a second-order polynomial equation. The ANOVA results of the dependent variables are shown in Table 4, while Table 5 shows the fit statistics values R^2^, predicted R^2^, adjusted R^2^, and adequate precision for each dependent variable analyzed.

The grape residue studied is the final residue obtained in the fermentation of the grapes for the winemaking process after it was fermented in the barrels and compressed to extract as much must as possible. Therefore, a drained bagasse residue is obtained, composed of skin, seeds, and stems. The whole bagasse residue, without separating the different parts, was milled to obtain the bagasse powder. The purpose of treating all the bagasse as a whole was to reduce the number of unit operations, simplifying the process and minimizing the final cost of production. The resulting bagasse powder after 24 h of drying at 50 °C showed a moisture content of 2.43 ± 0.15%. The water activity of the Garnacha bagasse powder measured was 0.32 ± 0.006. The lower water activity guarantees the stability of the bagasse powder through the storage time since bacteria usually require water activity values higher than 0.86 and fungi at least 0.6 [33,38].

### 3.1. Modeling for the Acid Hydrolysis of Garnacha Bagasse

The glucose concentration in the hydrolysates ranged from 3.24 to 5.40 g/L. Data fitted well to a two-factor interaction mathematical model, and no trials were detected as outliers in Cook’s distance test. By examining the sequential model sum of squares for both the partial and complete models, the mathematical model was chosen. The F-value of the model was 37.46, and the *p*-values of the model terms were significant (*p* < 0.05), implying that the glucose concentration was dependent on the hydrolysis conditions. There is only a 0.01% chance that an F-value this large could occur due to noise. The lack of fit F-value of 2.49 implied that it was not significant relative to the pure error. There is a 31.40% chance that a lack of fit F-value this large could occur due to noise.

The R^2^ value obtained was 0.97, which represents the relative predictive power of the model. However, it is important to note that this value alone does not determine whether the model can be applied to the entire population or only to the analyzed samples. To further evaluate the model’s performance, additional parameters such as adjusted R^2^, predicted R^2^ and adequate precision are considered. The adjusted R^2^ is a statistic that compares the goodness-of-fit among regression models with different numbers of independent variables. It considers whether additional variables contribute value to the model. If the model includes unnecessary variables, the adjusted R^2^ will decrease as the number of terms increases. On the other hand, the predicted R^2^ measures the model’s ability to accurately predict response values. When comparing the adjusted R^2^ and predicted R^2^, it is considered acceptable if the difference between them is within 0.2 units. This reasonable agreement indicates that the model performs well in predicting values. In summary, while the initial R^2^ value of 0.97 provides insight into the model’s predictive power, it is essential to consider additional parameters like adjusted R^2^ and predicted R^2^ to assess the model’s applicability and accuracy.

In this case, the difference between the predicted R^2^ (0.90) and the adjusted R^2^ (0.94) was less than 0.2, which is reasonable. Adequate precision is a signal/noise ratio. It compares the range of the predicted values at the design points to the average prediction error. Ratios greater than 4 indicate adequate model discrimination. The adequate precision obtained was 18.92, implying an adequate signal. 

The F-values of the terms allow for determining which component has a greater effect on the response. The F-values of the model terms indicated that the glucose concentration after hydrolysis treatment was mainly affected by the acid concentration (F-value = 72.56) and the temperature (F-value = 70.03) of the process, followed by the time (F-value = 52.03). The interaction between the components promoted a lower effect on the glucose concentration. Equation (4) predicts the glucose content of hydrolysate in terms of actual factors, where the levels should be specified in the original units for each factor. According to the initial setup, *A* refers to sulfuric acid concentration (% *w*/*w*), *B* is time (min), and *C* is the temperature (°C):

(4) $$Glucose\;\left(\frac{g}{L}\right)=-20.44+4.22\left(A\right)+0.06\left(B\right)+0.19\left(C\right)-2\cdot 10^{-3}\left(AB\right)-0.03\left(AC\right)-5\cdot 10^{-4}\left(BC\right)$$

**Figure 1:**
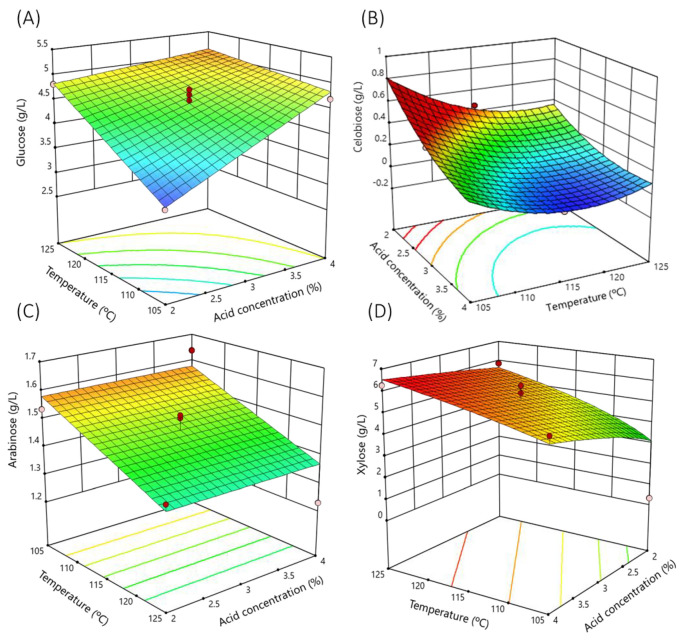
Response surface of the main sugars analyzed as a function of sulfuric acid concentration and temperature. Time fixed at 120 min: (**A**) glucose; (**B**) cellobiose; (**C**) arabinose; (**D**) xylose. Dots are the real values.

The 120 min hydrolysis surface response (Figure 1A) clearly shows how the glucose concentration depended mainly on the increase in acid concentration and temperature.

Data showed a low cellobiose content in the hydrolysates, obtaining a maximum of 0.48 g/L. Data fitted well to a quadratic mathematical model, and no trials were detected as outliers in Cook’s distance test. The model F-value of 23.38 implied the model was significant (*p*-value < 0.05). There was only a 0.14% chance that an F-value this large could occur due to noise. The content of cellobiose mainly depended on the acid concentration, the quadratic effect of the temperature, and the interaction between temperature and time. However, the interaction between acid concentration time, acid concentration temperature, and the quadratic effect of the concentration and time were also significant terms. 

The R^2^ value (0.98), the predicted R^2^ (0.75), and the adjusted R^2^ (0.94) were in reasonable agreement, and the adequate precision value (14.12) was higher than 4, implying an adequate signal. Equation (5) predicts the cellobiose content of hydrolysate in terms of actual factors where *A* is the sulfuric acid concentration (% *w*/*w*), *B* is time (min), and *C* is the temperature (°C):

(5) $$\begin{array}{c} Cellobiose\;\left(\frac{g}{L}\right)=29.49-1.79\left(A\right)-0.04\left(B\right)-0.43\left(C\right)+1\cdot 10^{-3}\left(AB\right)+8\\ \;\;\;\;\;\;\;\;\cdot 10^{-3}\left(AC\right)+2\cdot 10^{-4}\left(BC\right)+0.10\left(A^{2}\right)+2\cdot 10^{-4}\left(B^{2}\right)+2\cdot 10^{-3}\left(C^{2}\right) \end{array}$$

According to the response surface, considering the time of 120 min (Figure 1B), the cellobiose concentration in the hydrolysate mainly increased with the acid concentration.

The concentration of arabinose ranged between 0.66–1.64 g/L. In order to obtain a better predictive model, trial 1 was ignored since it was detected as an outlier in Cook’s distance test. Thus, data fit well to a linear model. However, a reduction of the model was necessary, as the statistical values indicated that the acid concentration had no significant effect. Furthermore, considering all parameters (acid concentration, temperature, and time) in the model, the predicted R^2^ was not as close to the adjusted R^2^ as one might normally expect (Table 5). By reducing the model and considering only the temperature and time variables, it was possible to obtain a good predictive model with good statistical values. The model F-value of 11.19 implied the model was significant (*p*-value < 0.05). There was only a 0.22% chance that an F-value this large could occur due to noise. The content of cellobiose depended on the linear effect of the temperature (F-value = 19.18). The R^2^ value was 0.67, and the predicted R^2^ (0.42) was in reasonable agreement with the adjusted R^2^ (0.61). Additionally, the adequate precision value (9.09) was higher than 4, implying an adequate signal.

Equation (6) predicts the arabinose content of hydrolysate in terms of actual factors:

(6) $$Arabinose\;\left(\frac{g}{L}\right)=2.63+4.36\cdot 10^{-4}\left(B\right)-1.05\cdot 10^{-2}\left(C\right)$$

According to the response surface (time 120 min) (Figure 1C), the arabinose concentration in the hydrolysate mainly increased with the increase of the temperature treatment.

The last monosaccharide analyzed was xylose, which ranged from 3.24 to 5.40 g/L. Data fitted well to a linear mathematical model after the data transformation into power with lambda 2.71 following the Box-Cox plot recommendation to obtain a better fit of the equation. Therefore, the model obtained was significant, with an F-value of 12.85 and a *p*-value < 0.05. There was only a 0.06% chance that an F-value this large could occur due to noise. By analyzing the F-values and *p*-values of the model terms, the acid concentration and temperature had a significant effect on the xylose concentration. The lack of fit F-value of 5.34 implied that it was not significant relative to the pure error. There is a 16.76% chance that a lack of fit F-value this large could occur due to noise.

The value of R^2^ was 0.78. The predicted R^2^ (0.61) was in reasonable agreement with the adjusted R^2^ (0.72). The adequate precision was higher than 4 (10.97), implying an adequate signal. Equation (7) predicts the xylose content of hydrolysate in terms of actual factors:

(7) $$Xylose^{2.71}\;\left(\frac{g}{L}\right)=-496.69+25.59\left(A\right)+0.30\left(B\right)+4.17\left(C\right)$$

According to the response surface (Figure 1D), the xylose concentration in the hydrolysate depended mainly on the temperature followed by the acid concentration. Figure 1D plots the variation of xylose concentration as a function of temperature and acid concentration at 120 min hydrolysis time. 

Our data were in agreement with previous data observed for the acid hydrolysis of the grape stalk, where maximum glucose (3.22 g/L), xylose (7.29 g/L), and arabinose (0.91 g/L) concentrations from the grape stalk after hydrolysis treatment with sulfuric acid at 3.5% for 60 min were reported [39]. In addition to the bagasse evaluated, which also included skins, pulp, and seeds, the monosaccharide profile after the hydrolysis was similar to those observed for grape stalks. The main reason is that most of the fermentable sugars in the bagasse were previously consumed by the yeasts during alcoholic fermentation in the winemaking process. In addition, bagasse was subjected to a draining and pressing process during wine production to extract all the monosaccharide-rich must. Higher glucose concentration was reported in studies in which whole grapes or grape juice were used directly [14,40].

On the other hand, the composition of potential inhibitor compounds such as acetic acid released from acetyl groups, furfural, and HMF generated by sugar dehydration was measured as a function of the hydrolysis conditions. Acetic acid concentration ranged 0.48–0.98 g/L. Originally, data fitted well to a quadratic mathematical model, and no trials were detected as outliers. The model obtained was significant with an F-value of 22.55, *p*-value < 0.05, and there was only a 0.16% chance that an F-value this large could occur due to noise. However, the difference between the predicted R^2^ (0.63) and the fitted R^2^ (0.93) was greater than 0.2. This observation could suggest the presence of a significant block effect or potentially highlight an issue with the model. For this reason, a reduction of the model has been taken into account to improve the statistical fits. The F-values and *p*-values of the model terms indicated that the interaction time–temperature and the quadratic effect of the temperature had no significant effect on the acetic acid concentration. Thus, these parameters were removed from the model. The reduced model had an F-value of 67.14 and a *p*-value < 0.0001 (Table 4). Statistical analysis indicated that the acetic acid concentration mainly depended on the temperature (F-value 256.01) of the hydrolysis, followed by the sulfuric acid concentration (F-value 81.91). The lack of fit F-value of 5.71 implied there was a 15.45% chance that a lack of Fit F-value this large could occur due to noise.

The R^2^ value of the reduced model was 0.99, and the predicted R^2^ (0.97) was in reasonable agreement with the adjusted R^2^ (0.90). The adequate precision was 25.00, indicating an adequate signal.

Equation (8) predicts the acetic acid content of hydrolysate in terms of actual factors:

(8) $$Acetic\;acid\;\left(\frac{g}{L}\right)=-3.79+1.136\left(A\right)+0.01\left(B\right)+0.03\left(C\right)-4.6\cdot 10^{-4}\left(A\cdot B\right)-3.5\cdot 10^{-3}\left(A\cdot C\right)-0.09\left(A^{2}\right)-2.9\cdot 10^{-5}\left(B^{2}\right)$$

**Figure 2:**
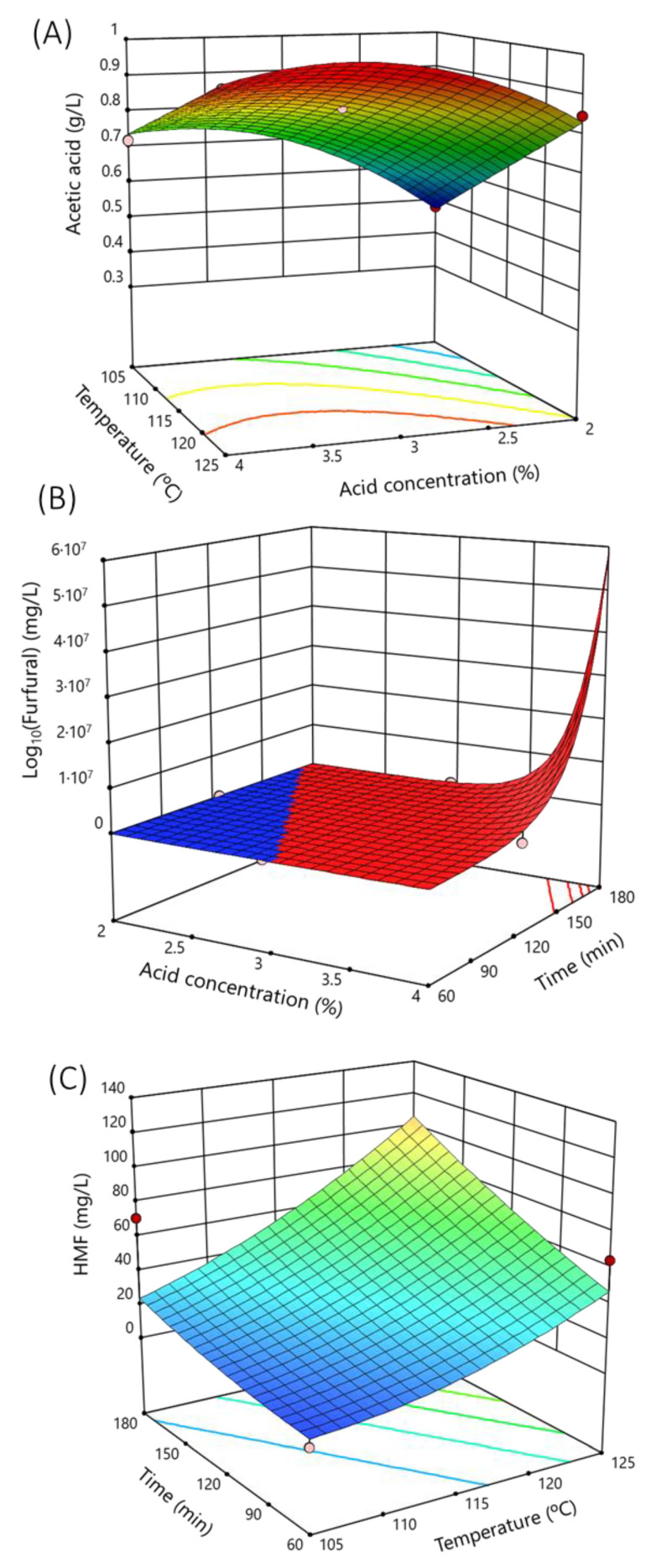
Response surface of the main microbial growth inhibitors analyzed as a function of main hydrolysis variables (sulfuric acid concentration, temperature, or time). (**A**) Response surface of acetic acid at 120 min of hydrolysis time. (**B**) Response surface of furfural at a hydrolysis temperature of 125 °C. (**C**) Response surface of 5-(hydroxymethyl)-2-furaldehyde (HMF) at 3% sulfuric acid concentration. Dots are the real values.

Figure 2A shows the response surface of the acetic acid concentration at 120 min, showing the strong dependence on temperature, followed by the acid concentration. 

The maximum furfural concentration was 146.35 mg/L. The Box-Cox plot recommended transforming the data to base 10 logarithms to obtain a better fit of the equation. As a result, data fitted well to a linear mathematical model and no trials were detected as outliers. The model F-value of 6.39 and *p*-value < 0.05 pointed out the model was significant. There was only a 0.18% chance that an F-value this large could occur due to noise. Note that the F-values and *p*-values of the model terms suggested that firstly, the sulfuric acid concentration and then time were the parameters with higher effect on the furfural production.

The value of R^2^ was 0.64, but the value of the predicted R² (0.30) was not as close to the adjusted R² (0.54) as one might normally expect. This may indicate a large block effect. However, the adequate precision was higher (7.65), indicating an adequate signal, and this model can be used to navigate the design space.

Equation (9) forecasts the furfural concentration (mg/L) of hydrolysate in terms of actual factors:

(9) $$Log_{10}\left(Furfural\right)\left(\frac{mg}{L}\right)=-21.71+2.28\left(A\right)+0.02\left(B\right)+010\left(C\right)$$

Hence, the furfural concentration in the hydrolysate depended on the sulfuric acid concentration and the set time of the process, as the response surface graph (Figure 2B) shows for 125 °C.

In the case of HMF, the maximum concentration reached was slightly lower than furfural, being up to 124.79 mg/L. The Box-Cox plot recommended transforming the HMF concentration data to a square root of the HMF concentration to better fit. As a result, data fitted well to a linear mathematical model, and no trials were detected as outliers. The model F-value of 7.36 and *p*-value < 0.05 pointed out the model was significant. There was only a 3.46% chance that an F-value this large could occur due to noise. Unlike the effect observed for furfural, the content of HMF depended on the temperature and time of the hydrolysis. 

The value of R^2^ (0.67) and the predicted R² (0.30) was not as close to the adjusted R² (0.58) as one might normally expect. This may indicate a large block effect. However, the adequate precision was higher (8.49), indicating an adequate signal, and this model can be used to navigate the design space.

Equation (10) estimates the HMF concentration (mg/L) of hydrolysate in terms of actual factors:

(10) $$\sqrt{HMF}\left(\frac{mg}{L}\right)=-33.67+1.02\left(A\right)+0.03\left(B\right)+0.28\left(C\right)\;$$

Figure 2C represents the response surface of HMF production at a constant acid concentration of 3%. Note that the HMF production was dependent on the set time and temperature of the heat treatment.

Regarding the presence of antioxidant compounds after hydrolysis treatment, the TPC was measured. Data showed that the TPC of the hydrolyzed bagasse extract remained between 7.23–12.72 mg GAE/g dried Garnacha Tintorera powder. The ANOVA analysis indicated that the model was not significant (*p*-value > 0.05). Hence, the parameters selected (sulfuric acid concentration, temperature, and time) in the range studied had no significant effect on the TPC obtained in the bagasse hydrolysate.

Considering the results, the TPC of aqueous bagasse extract without hydrolysis treatment was measured. For this purpose, an aqueous bagasse extract was prepared with the same solid/liquid ratio (1:10) under vigorous stirring for 1 h at 30 °C. Under these conditions, the bagasse aliquot had a TPC of 4.39 mg GAE/g dried Garnacha Tintorera powder. Comparing the results by hydrolysis conditions, it was possible to obtain more than double the TPC concentration. Additionally, the TPC of the hydrolyzed bagasse extract was higher than that reported by other works using other extraction methods, such as ohmic heating treatment (3.0–9.8 mg GAE/g bagasse) [5]. On the other side, it was reported a higher TPC yield (22.61 mg GAE/g) from bagasse juice in ethanol (50%) extraction with a relation of 50 mL per gram of bagasse [41].

### 3.2. Optimization of the Acid Hydrolysis of Grape Bagasse

According to the mathematical models obtained for each studied variable, the optimization was carried out by establishing the purpose of the optimization (minimize, maximize, or keep in range the parameter) and the priority criteria for each variable (Table 6). The priority criteria were scaled between 1 (lowest priority) and 5 (highest priority. The criteria have been established considering the effect of high acid concentration and treatment time, which can lead to the formation of HMF and furfural due to the degradation of complex polysaccharides. In this dilute hydrolysis, inhibitory compounds are formed by hydrolysis of hemicellulose to xylose and subsequent dehydration to acetic acid, furfural, and HMF [39].

According to the established criteria and the predictive models, the hydrolysis condition selected was 125 °C for 60 min with a diluted sulfuric acid concentration of 2% (*w*/*w*). Under the selected conditions, the mathematical models predicted that the concentrations of the hydrolysates are as follows: 4.61 g/L glucose, 0.37 g/L cellobiose, 1.60 g/L arabinose, acetic acid 0.67 g/L, xylose 5.33 g/L, furfural 2.5 mg/L, HMF 0.3 mg/L, and TPC 10.08 mg GAE/g dried bagasse. These conditions were used in the next sections.

### 3.3. Sustainable Culture Media with Phenolic Compounds

Garnacha grape bagasse hydrolysates were tested as culture media for BC production in static operation [35]. The low monosaccharides concentration on the bagasse hydrolysates resulted in the production of a residual, weak, and non-homogeneous BC film [35].

However, the hydrolysate of grape bagasse could enhance the nutrients and micronutrient profile of the culture media with another carbon source. The high content of phenolic and other antioxidant components or micronutrients may boost microbial growth and yield [40,42]. The operational conditions of the optimized grape bagasse hydrolysis are close to those obtained in previous studies for potato hydrolysis [6]. Non-commercial potato waste hydrolysate showed a rich glucose concentration (up to 74.75 g/L) after hydrolysis treatment at 130 °C for 30 min with an acid solution of sulfuric acid at 3% and a solid/liquid ratio of 1:10 (*w*/*w*) [6].

**Figure 3:**
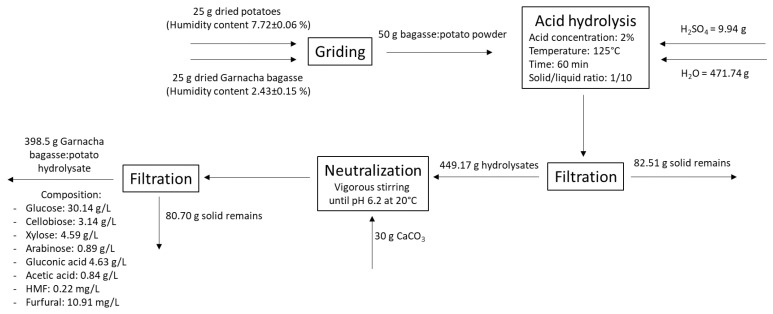
Overall process proposed for the pre-treatment and sulfuric acid hydrolysis of Garnacha Tintorera grape bagasse–discarded potatoes powder mixture.

Therefore, mixing non-commercial potato waste with grape bagasse could be a promising alternative to obtain a low-cost culture media with high glucose concentration and optimal nutrients and micronutrient profiles for biotechnology fermentations. Culture media based on discarded potato and Garnacha grape bagasse was proposed with a ratio of 50:50 (*w*/*w*). The hydrolysis treatment was carried out at 125 °C for 60 min with 2% sulfuric acid concentration, according to the optimized hydrolysis for Garnacha grape bagasse. The overall process with the mass balance is shown in Figure 3. 

From an initial mixture of 25 g of dry and powdered bagasse and 25 g of potato, 471.74 g of water and 9.94 g of sulfuric acid were added and subjected to hydrolysis under the conditions set out in the optimization process. The hydrolysate was neutralized with CaCO_3_ until pH 6.2 and subsequently filtered to remove solid residues. Finally, 398.5 g of aqueous extract was obtained, with a giving liquid yielding 84.5%. Afterwards, this extract enriched with yeast extract was evaluated as a culture medium to produce BC and gluconic acid, as shown in the next section.

### 3.4. Co-Production of Bacterial Cellulose and Gluconic Acid

**Figure 4:**
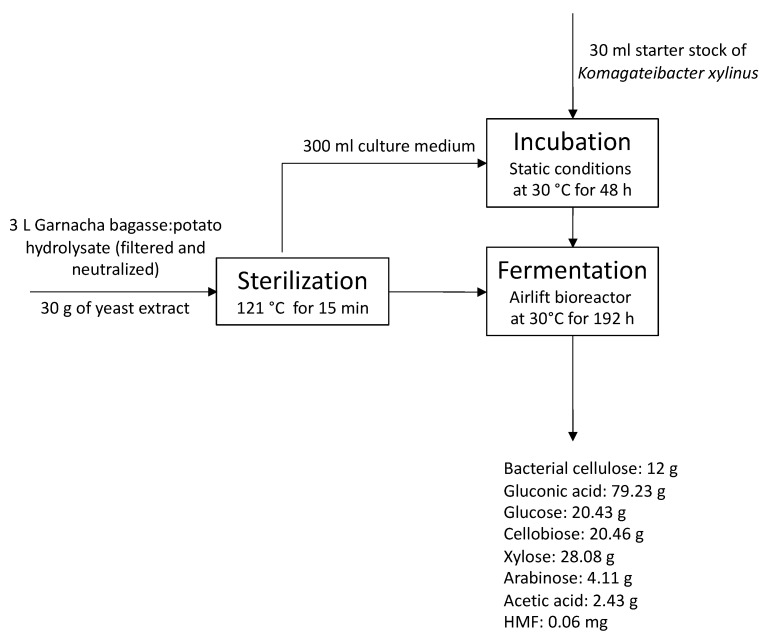
Overall process proposed for the co-production of bacterial cellulose and gluconic acid by *Komagataibacter xylinus* fermentation of a low-cost alternative culture medium based on Garnacha Tintorera grape bagasse–discarded potatoes.

The hydrolysate of grape bagasse and potato with a high content of phenolic compounds was tested for the co-production of BC and gluconic acid in an airlift bioreactor (Figure 4). The airlift bioreactor allows for increased oxygen delivery using a lower power supply than a stirred tank bioreactor [43]. Additionally, this reactor produces less shear stress than a stirred tank [43].

The neutralized culture media were used for the co-production of BC and gluconic acid by the fermentation of *K. xylinus* for 8 days to study the suitability of the developed low-cost culture media and confirm that the current concentration of microbial growth inhibitors did not limit its application. The fermentation time was established on the basis of previous experience and the monitoring of glucose concentration, gluconic acid, and pH every 24 h.

The initial composition of the resulting bagasse–potato culture medium was: 30.14 g glucose/L, 3.14 g cellobiose/L, 4.59 g xylose/L, 0.89 g arabinose/L, 4.63 g gluconic acid/L, 0.84 g acetic acid/L, 0.22 mg HMF/L and 10.91 mg furfural/L. Results confirmed that the hydrolyzed potato starch increased the glucose concentration in the current culture media by more than 6-fold compared with the pure Garnacha grape bagasse hydrolysates [6,44]. The glucose concentrations measured are in range with data previously obtained for pure hydrolysates of potato [6]. The studied culture medium showed higher glucose concentration than those reported for the acid hydrolysis of potato peelings [45] due to the highest amount of starch using the whole potato instead of just the peel. The cellobiose, xylose, and arabinose were products derived from the hydrolysis of pectic and hemicellulosic polysaccharides of the grape bagasse, as indicated by the bagasse hydrolysis data.

**Figure 5:**
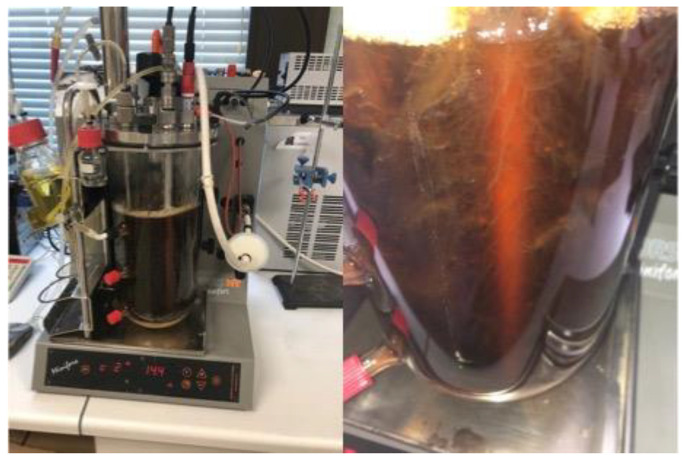
Bacterial cellulose growth in an airlift fermenter with a culture medium based on Garnacha Tintorera grape bagasse and discarded potatoes.

Figure 5 shows the airlift bioreactor with a detail of the BC produced as fibers. A sample of the fermentation medium was taken every 24 h to analyze the content of the main compounds. 

**Figure 6:**
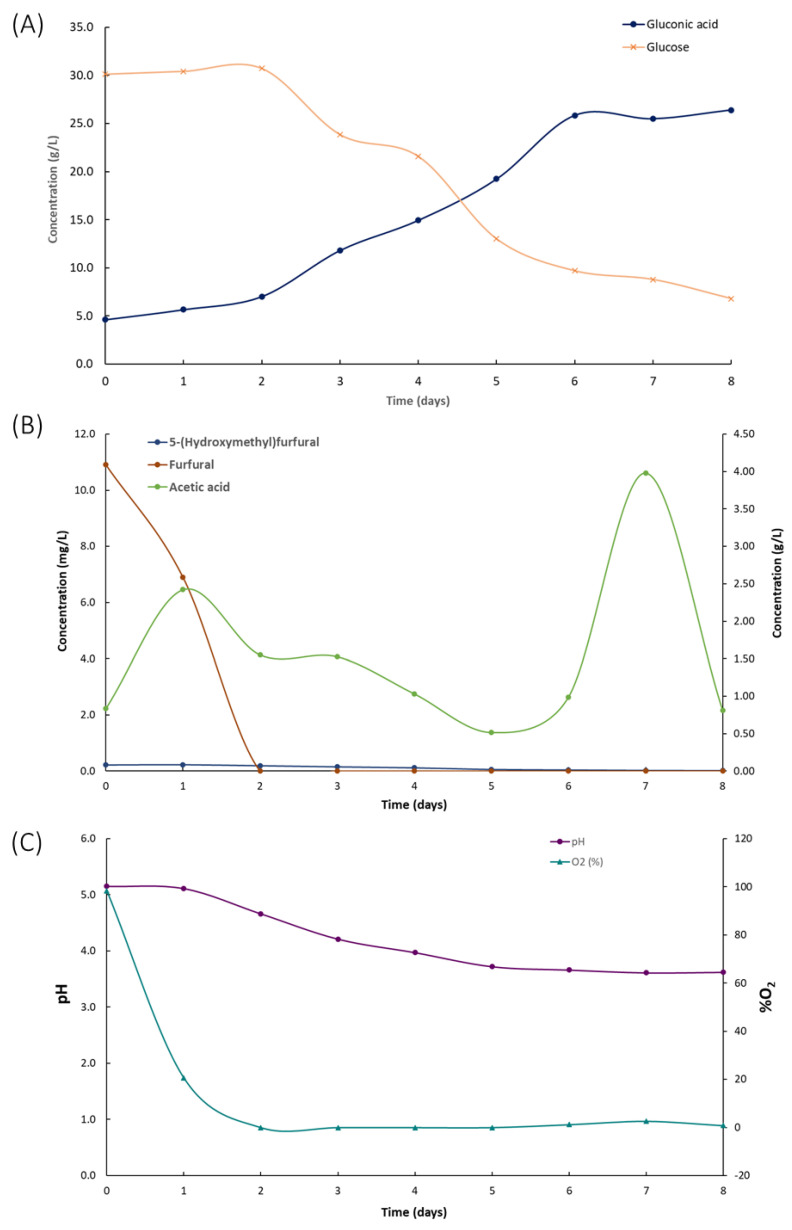
Kinetics in the composition of the culture medium by the fermentation of *Komagateibacter xylinus* in a culture medium based on Garnacha Tintorera grape bagasse and discarded potatoes. (**A**) Glucose and gluconic acid concentrations; (**B**) microbial growth inhibitors; (**C**) pH and dissolved O_2_.

Figure 6A illustrates the concentration of glucose and gluconic acid during fermentation. A 2-day lag phase was observed before glucose consumption began. Glucose is the primary carbon source in the medium, and its decline was attributed to bacterial growth and BC production. Furthermore, the time with the lower glucose concentrations matched with the highest production of gluconic acid, reaching a concentration of 26.41 g/L. The ability of *K. xylinus* to produce gluconic acid has been previously documented in the literature [30]. The glucose dehydrogenase located in the cytoplasmic membrane of *K. xylinus* oxidizes glucose to gluconic acid. The conversion of glucose into gluconic acid resulted in a strong decrease in BC production [30]. 

Additionally, gluconic acid promotes a significant decrease in the pH of the culture medium, which inhibits bacteria activity and BC synthesis [30,46,47]. Despite the fact that *K. xylinus* is able to assimilate gluconic acid to continue producing BC, BC production decreases drastically, and it may not be profitable to continue fermentation in static conditions [35]. One way to address the limit on BC production and reduce its cost is to identify optimal conditions for maximum production of both BC and gluconic acid. This would enable the simultaneous production of two valuable and commercially significant products in a single fermentation process.

Kinetics of the microbial growth inhibitors are shown in Figure 6B. The initial concentration of furfural (10.91 mg/L), HMF (0.22 mg/L), and acetic acid (0.84 g/L) as non-desirable by-products of the hydrolysis did not inhibit the bacterial growth. Previous work reported a significant inhibitory effect of HMF and furfural on the activity of *K. xylinus* with a concentration of 2 g/L HMF and 0.4 g/L furfural [48]. It should be noted that the activity of *K. xylinus* promoted the decrease of furfural and HMF. The furfural concentration drastically decreased in 2 days until 0 mg/L. To a lesser extent, the HMF concentration decreased up to 0.02 mg/L at the end of the fermentation (8 days). The results showed that *K. xylinus* bacteria were capable of consuming HMF and furfural at the evaluated concentration levels, consistent with findings reported in other studies [35]. These findings suggest that *K. xylinus* could be used in bioremediation efforts to address HMF- and furfural-contaminated environments, potentially providing a cost-effective and sustainable solution for environmental cleanup. This application should be evaluated in future studies.

After 8 days of fermentation, 4 g dried BC/L with a productivity of 0.021 g/L·h were obtained (Figure 4). In static fermentation conditions, using Petri dishes with 25 mL of the proposed culture broth, similar BC concentration values were achieved (4 g dried BC/L) [35]. Despite observing similar BC concentration levels, the main difference between static and airlift fermentation was the efficacy and yield values. In the airlift bioreactor, the BC production by the fermentation of *K. xylinus* in Garnacha bagasse–potato culture broth showed a BC efficiency of 0.37 g/g and a yield of 0.47 g/g. In static conditions, BC efficacy was 0.12 g/g, and the yield was 0.13 g/g [35]. This implies that similar BC concentration values can be achieved in an airlift fermenter as in static conditions by consuming less glucose. As reflected by the data, under static conditions at day 7 of fermentation, the glucose concentration in the medium is approximately 1 g/L, being 0 g/L at day 8 [35]. However, in the airlift fermenter, at the end of fermentation, there was still a glucose concentration of 6.81 g/L.

Regarding gluconic acid, 26.41 g gluconic acid/L with a productivity of 0.14 g/L·h, an efficiency of 0.93 g/g, and a yield of 0.72 g/g were measured. Airlift fermentation allowed an increase in gluconic acid production 2,68 times compared to static fermentation (9.86 g/L). Note that under static conditions, to reach BC concentrations similar to airlift, it was necessary for the bacteria to consume gluconic acid as a carbon source, which significantly reduced the availability of this compound. However, through the aeration process generated in the airlift, it is possible to reach maximum values of both BC and gluconic acid with lower consumption of glucose as a carbon source, which resulted in higher efficiency and yield values.

The BC yield reached in the present work was higher than those observed in the literature; meanwhile, productivity was higher or similar. For example, fermentations with winery wastes or grape juice with higher initial glucose content reported production of 4 g/L in 18 days (productivity of 0.009 g/L·h) using grape pomace and corn steep liquor [11]. H-S medium-enriched white grape bagasse yielded 1.2 g/L after 14 days at 28 °C (productivity of 0.004 g/L·h) [13]. Hydroxylated potato peel waste resulted in BC production up to 2.61 g/L after 96 h of incubation (productivity of 0.027 g/L·h) [45]. In the airlift bioreactor, the BC production was 3.8 g after 67 h using a corn steep liquor-fructose medium, and a modified airlift bioreactor allowed to obtain the highest harvest (10.4 g/L) [49], but in these cases, no production of gluconic acid was evaluated.

The pH and O_2_ partial pressure through the fermentation are shown in Figure 6C. The sharp decrease in pH (from 5.15 to 3.66) and O_2_ partial pressure (from 98.3% to 0%) confirms high bacterial activity during this period and the high demand for oxygen. Furthermore, by introducing phenolic compounds into the culture medium during the synthesis of BC, these compounds were successfully integrated into the polymeric matrix. This incorporation of phenolic compounds led to the development of BC with antioxidant capacity, measuring at 1.3 mg GAE/g dried BC, similar to the results observed in static fermentation [35]. This functionalization of the synthesized polymer enhances its potential for future applications.

Comparing the results of gluconic acid production with previous works, the gluconic acid production obtained was lower than those obtained by the fermentation of *Aspergillus niger* in alternative culture media. For instance, banana must and grape must be tested as carbon sources for the production of gluconic acid by *A. niger* [23,24]. After 8 days, the gluconic acid produced by *A. niger* ranged between 60–70 g/L for a culture media with an initial glucose concentration of 120 g/L. Although the gluconic acid content was lower in this study compared to fungal fermentations, it is noteworthy that our approach enabled the simultaneous production of two commercially valuable products, BC and gluconic acid.

## 4. Conclusions

In this work, the feasibility of a culture medium based on Garnacha Tintorera grape bagasse (as a source of phenolic compounds and other micronutrients) and waste potatoes (as a main source of glucose) for the co-production of BC and gluconic acid by the fermentation of *K. xylinus* in an airlift bioreactor has been successfully evaluated.

Acid hydrolysis of bagasse from Garnacha Tintorera grapes has been studied to obtain an extract with a higher content of phenolic compounds than aqueous extract, keeping the levels of microbial growth inhibitor by-products such as furfural, HMF, and acetic acid low. Statistical analysis suggested that acid concentration and temperature were the operational variables with the main effect on the composition of the hydrolysates. The optimization proposed a 2% sulfuric acid concentration for 60 min at 125 °C as optimal hydrolysis conditions. Under these hydrolysis conditions, it was possible to obtain more than twice the concentration of TPC (10.08 mg GAE/g dried bagasse) compared to a simple aqueous extraction (4.39 mg GAE/g dried bagasse) or to other methods previously evaluated.

These hydrolysis conditions also allowed the hydrolysis of potato starch to obtain high glucose concentrations and keep concentrations of furfural, HMF, and acetic acid low. Hence, the culture medium resulted from the blend of grape bagasse and potato hydrolysates (50:50 *w/w* ratio) was a low-cost culture medium rich in micronutrients and a carbon source for the production of BC and gluconic acid from agri-food industry waste using an airlift bioreactor. The gluconic acid production increased 2.68 times compared to static fermentation.

On the other hand, a drastic reduction of furfural and HMF levels has been observed due to the activity of *K. xylinus*. This ability of the microorganism to consume these by-products originating from hydrolysis processes and microbial growth inhibitors deserves special attention and should be analyzed in depth in future work. Since depending on the HMF and furfural concentration levels at which the activity of the bacterium is not affected and can reduce their concentration, it may open the door to new bioremediation applications of *K. xylinus*.

In summary, this research demonstrates the promising potential of utilizing waste materials, specifically Garnacha Tintorera bagasse and potato residues, as sustainable substrates for the co-production of valuable bioproducts, such as bacterial cellulose and gluconic acid. These findings not only contribute to the development of eco-friendly and economically viable processes but also emphasize the importance of exploring innovative approaches for waste utilization in the context of biotechnology and sustainable resource management.

## Figures and Tables

**Table 1:** Variables involved in the Box–Behnken experimental design for sulfuric acid hydrolysis of dried grape bagasse (Garnacha Tintorera).

Factors ^(1)^	Nomenclature	Units	Variation Levels
Sulfuric acid	A	g/L	2, 3, 4
Time	B	min	60, 120, 180
Temperature	C	°C	105, 115, 125
**Dimensionless**			
Sulfuric acid	X_1_		−1, 0, 1
Time	X_2_		−1, 0, 1
Temperature	X_3_		−1, 0, 1
**Response ^(2)^**			
Glucose		g/L	
Celobiose		g/L	
Arabinose		g/L	
Acetic acid		g/L	
Xylose		g/L	
Furfural		mg/L	
HMF ^(3)^		mg/L	
TPC ^(4)^		mg GAE/g dried bagasse	

^(1)^ Independent variables, ^(2)^ Dependent variables, ^(3)^ 5-hydroxymethyl furfural, ^(4)^ Total phenolic content.

**Table 2:** Box–Behnken experimental design with three factors and three levels for the surface response study of the acid hydrolysis of grape bagasse.

Run	Sulfuric Acid	Time	Temperature
	%	min	°C
1	3	180	125
2	4	60	115
3	2	120	125
4	3	120	115
5	3	60	105
6	4	120	125
7	3	120	115
8	2	180	115
9	2	120	105
10	3	120	115
11	3	60	125
12	4	120	105
13	2	60	115
14	4	180	115
15	3	180	105

**Table 3:** Responses obtained for the concentration of the dependent variables: glucose, cellobiose, arabinose, xylose, acetic acid, 5-hydroxymethyl furfural (HMF), furfural (F), and total phenolic content (TPC) following the Box–Behnken experimental design.

Run	Glucose	Cellobiose	Arabinose	Xylose	Acetic Acid	HMF	F	TPC
	g/L	g/L	g/L	g/L	g/L	mg/L	mg/L	mg GAE *
1	4.97	0.39	0.66	5.87	0.88	70.39	1.26	12.35
2	4.72	0.00	1.45	5.17	0.75	24.22	2.14	9.65
3	4.80	0.32	1.39	5.86	0.85	124.79	0	11.68
4	4.58	0.00	1.43	5.66	0.84	31.53	8.68	9.68
5	3.39	0.42	1.48	3.44	0.56	0.22	0	12.72
6	5.02	0.26	1.23	6.26	0.94	67.85	146.35	11.31
7	4.79	0.00	1.53	6.01	0.86	23.23	6.85	10.59
8	4.57	0.27	1.52	5.48	0.76	15.24	3.48	9.64
9	3.24	0.48	1.53	0.31	0.48	0.31	0	10.30
10	4.69	0.07	1.52	6.01	0.85	30.52	10.08	8.74
11	4.81	0.11	1.40	6.12	0.88	64.19	32.03	12.32
12	4.69	0.10	1.64	5.29	0.72	13.99	1.39	11.00
13	3.50	0.46	1.49	3.54	0.48	0.57	0	9.45
14	5.40	0.12	1.52	6.11	0.77	58.77	43.68	12.51
15	4.63	0.18	1.61	4.77	0.64	70.87	2.53	7.23

* mg gallic acid equivalent/g dried Garnacha Tintorera bagasse.

**Table 4:** Analysis of variance (ANOVA) for each of the study-dependent variables. HMF is 5-hydroxymethyl furfural, and TPC is total phenolic content.

	**Glucose**		**Cellobiose**		**Arabinose**		**Xylose**	
**Source**	**F-Value**	***p*-Value**	**F Value**	***p*-Value**	**F Value**	***p*-Value**	**F Value**	***p*-Value**
Model	37.46	<0.0001	23.38	0.0014	11.19	0.0022	12.85	0.006
A ^(1)^	72.56	<0.0001	72.46	0.0004			9.28	0.0111
B ^(2)^	52.03	<0.0001	0.0292	0.8711	1.19	0.2995	4.65	0.0541
C ^(3)^	70.03	<0.0001	0.5828	0.4797	19.18	0.0011	24.61	0.0004
AB	1.51	0.2547	12.86	0.0158				
AC	16.12	0.0039	12.65	0.0163				
BC	12.54	0.0076	36.09	0.0018				
A²			20.11	0.0065				
B²			14.60	0.0124				
C²			51.08	0.0008				
Lack of Fit	2.49	0.314	1.14	0.4984	1.37	0.4918	25.58	0.1676
	**Acetic Acid**		**HMF**		**Furfural**		**TPC**	
**Source**	**F-value**	***p*-value**	**F value**	***p*-value**	**F value**	***p*-value**	**F value**	***p*-value**
Model	67.14	<0.0001	7.36	0.0056	6.39	0.0091	1.27	0.3686
A	81.91	0.0001	1.89	0.1966	12.55	0.0046	0.6425	0.446
B	8.08	0.0295	5.72	0.0357	4.17	0.0658	0.3256	0.5839
C	256.01	<0.0001	14.48	0.0029	2.45	0.1459	2.29	0.1686
AB	2.64	0.1552					0.798	0.3978
AC	7.68	0.0323					0.1287	0.7291
BC							3.41	0.1021
A²	42.54	0.0006						
B²	47.81	0.0005						
C²								
Lack of Fit	5.71	0.1545					3.12	0.2622

^(1)^ Sulfuric acid concentration; ^(2)^ Time; ^(3)^ Temperature.

**Table 5:** Fit statistics of the model for each analyzed dependent variable. HMF is 5-hydroxymethyl furfural.

	Glucose	Cellobiose	Arabinose	Xylose	Acetic Acid	HMF	Furfural
R²	0.97	0.98	0.67	0.78	0.99	0.67	0.64
Adjusted R^2^	0.94	0.94	0.61	0.72	0.97	0.58	0.54
Predicted R^2^	0.90	0.75	0.42	0.61	0.90	0.30	0.30
Adeq Precision	18.92	14.12	9.09	10.97	25.00	8.49	7.65

**Table 6:** Optimization parameters for the acid hydrolysis of dried grape bagasse (Garnacha Tintorera). HMF is 5-hydroxymethyl furfural, and TPC is total phenolic content.

	Goal	Importance
Sulfuric acid	minimize	3
Time	minimize	3
Temperature	In range	
Glucose	maximize	5
Cellobiose	maximize	2
Arabinose	maximize	2
Acetic acid	minimize	3
Xylose	maximize	2
Furfural	minimize	2
HMF	minimize	2
TPC	maximize	4

## Data Availability

The data presented in this study are available on request from the corresponding author.
